# Use of proton pump inhibitor may be associated with progression of cerebral small vessel disease

**DOI:** 10.1371/journal.pone.0279257

**Published:** 2022-12-21

**Authors:** Min Kyoung Kang, Jung Hwan Shin, Tae Jung Kim, Ji Sung Lee, Byung-Woo Yoon, Sang-Bae Ko

**Affiliations:** 1 Department of Neurology, Uijeongbu Eulji Medical Center, Uijeongbu, Gyeonggi, Republic of Korea; 2 Department of Neurology, Seoul National University Hospital, Seoul, Republic of Korea; 3 Clinical Research Center, Asan Medical Center, Seoul, Republic of Korea; 4 Department of Neurology, Seoul National University College of Medicine, Seoul, Republic of Korea; Ehime University Graduate School of Medicine, JAPAN

## Abstract

Proton pump inhibitors (PPIs) are widely used for the treatment of gastrointestinal diseases. However, recent studies have shown that chronic PPI use is associated with the progression of endothelial senescence and cerebrovascular diseases. We hypothesized that PPI users might be vulnerable to fast progression of cerebral small vessel disease (SVD) with cumulative effects. Four hundred and eleven patients, who underwent brain magnetic resonance imaging, more than twice between January 2010 and December 2016 were screened. Patients aged < 50 years, and those who had concomitant diseases that might affect the progression of cerebral SVD were excluded. Baseline characteristics were collected. We evaluated the severity of SVD using the Fazekas score, the number of cerebral microbleeds (CMBs), and assessed the progression of SVD or CMBs based on the cumulative dose of PPIs. Among the included patients (N = 137), 39 were PPI ever-users. Univariate Cox regression analysis showed that PPI use was independently associated with the progression of Fazekas score only in the deep white matter hyperintensities (WMH) (hazard ratio [HR] 2.891, 95% confidence interval [CI] 1.210–6.906, P = 0.017). In multivariate Cox regression analysis, long-term PPI use was associated with a progression of Fazekas score in the deep WMH (HR 3.453, 95% CI 1.027–9.475, P = 0.045). However, PPI use was not associated with the progression of CMB. The present study results suggest that long-term use of PPIs is associated with the progression of deep cerebral WMH. Further research is needed using a large number of patients to validate this relationship.

## Introduction

Proton pump inhibitors (PPIs) are potent gastric acid-suppressing agents that have been used for the treatment of acid-peptic disorders. PPIs are often prescribed in stroke patients to prevent or treat gastrointestinal bleeding while using antiplatelet agents [1, 2]. The short-term use of PPIs is generally well-tolerated. However, there are concerns related to their chronic use, such as malabsorption syndrome, nutritional deficiency, bone fractures, and dementia [3–5]. A recent report showed that chronic PPI use was associated with an increased risk of a major cardiovascular event [6]. In addition, an experimental study revealed that chronic PPI use prompted vascular endothelial senescence, which could be the underlining mechanism for vascular complications from PPI use [7–9].

Cerebral white matter hyperintensities (WMH) and cerebral microbleeds (CMB) are frequent in elderly hypertensives, and are generally regarded as surrogate markers for cerebral vascular aging [10, 11]. Therefore, we hypothesized that PPI users might be more vulnerable to faster progression of WMH and CMB than PPI non-users with cumulative effects.

## Materials and methods

### Study subjects

A total of 7,002 patients (aged ≥ 18 years) who underwent brain magnetic resonance imaging (MRI) and magnetic resonance angiography (MRA) at the neurology outpatient clinic were screened between January 2010 and December 2016. Among them, 411 patients underwent follow-up brain MRI during the study period. We excluded patients with the following conditions: 1) age < 50 years (N = 64); 2) a history of an ischemic stroke or transient ischemia attack within 90 days (N = 106) or a history of hemorrhagic stroke, including subarachnoid hemorrhage and intracerebral hemorrhage (N = 19); 3) a moderate to severe stenosis (≥ 50%) in the intracranial arteries and their branches (N = 34); 4) arteriovenous malformation (N = 4); 5) underlying diseases that might affect the progression of white matter diseases (systemic or cerebral vasculitis (N = 29), history of intracranial radiation therapy (N = 2), or head trauma (N = 3)); and 6) intra-arterial procedures including glue or coil embolization and stent insertion (N = 6) [10]. We also excluded patients who had been treated with PPIs before the study period because the exact amount of exposure to PPI was not quantified (N = 7). As a result, a total of 137 patients were included in the final analysis (S1 Fig). Included patients were categorized as PPI ever users and PPI never users, based on the prescription information [4]. PPI ever users were further stratified into two groups: long-term users (prescribed PPI more than six months continuously) and PPI intermittent users (prescribed PPI less than six months continuously) [12]. The measurement of PPI use was calculated using the cumulative defined daily doses (DDD) according to the World Health Organization Collaborating Centre for Drug Statistics and Methodology [13]. The difference in the progression rate of WMH and CMB was compared between both groups. The Institutional Review Board of Seoul National University Hospital approved this study with waivers of the consent (IRB no. H-1911-074-1078).

### Neuroimaging: *Analysis of WMH and CMB*

Progression of WMH and CMB was assessed using the difference between baseline and follow-up brain imaging during the study period. Brain MRI was performed using a 1.5-T MR scanner with an eight-channel head coil (Philips Ingenia; Philips, Best, the Netherlands) or a 3.0-T MR imager with a 16-channel head coil (Verio; Siemens, Erlangen, Germany). WMH was discriminated from perivascular spaces or lacunar infarcts and subcategorized as periventricular and deep WMH [14]. CMB was also counted as previously described [15]. Periventricular and deep WMH were separately evaluated to clarify the impact of PPI on cerebral small vessel disease (SVD) in a specific area. Progression of SVD was defined as an aggravation of more than one grade of Fazekas score or development of more than one CMB [16, 17]. Visual gradings and counting were performed independently by two raters (MKK and JHS), with discrepancies being resolved in consultation with a third reviewer (SBK).

### Statistical analysis of the clinical data

Group comparisons were made using the t-test for continuous variables and Chi-square test for categorical variables. Nonparametric tests were performed using the Mann–Whitney U test. Multivariate Cox regression analysis was performed to estimate the risk for the progression of WMH and CMB. Variables that were confounding factors for the progression of SVD and associated with a significance level of *P*<0.1 in the univariate analyses were adjusted for multivariate analysis. Bonferroni corrected pairwise comparisons were conducted post hoc analysis. The level of statistical significance was set at P<0.05. All statistical analyses were performed by a professional medical statistician (JS Lee) using SPSS software (version 25.0, SPSS Inc., Chicago, IL, USA) and Matlab (Matlab Version R2019a, The MathWorks Inc., Natick, USA) with two-sided significance set at 0.05.

## Results

### Baseline characteristics

A total of 137 patients were included in the analysis. Indications for MRI follow-up were as follows: 1) checking the progression of known intracranial artery stenosis or aneurysm (n = 54, 39.4%), 2) dizziness (n = 48, 35.0%), 3) headache (n = 21, 15.3%), 4) memory decline (n = 9, 6.6%), and 5) syncope (n = 5, 3.7%). Baseline demographics and clinical information are presented in Table 1. Among the subjects included, 39 patients (28.5%) were PPI ever users. The PPIs included lansoprazole (n = 16, 41.0%), rabeprazole (n = 12, 30.8%), omeprazole (n = 6, 15.4%), pantoprazole (n = 3, 7.7%), and esomeprazole and dexlansoprazole (n = 2, 5.1%). The median cumulative DDD of PPI was 90 (interquartile range, 28–365). PPI ever users were younger (65.6 ± 6.1 years vs. 67.1 ± 9.1 years, P = 0.002) and were more frequently on antiplatelet drugs than PPI never users (48.7% vs. 29.6%, P = 0.034). There were no significant differences in terms of other vascular risk factors, concomitant medications, including calcium channel blockers, angiotensin-converting enzyme inhibitors, angiotensin receptor blockers, diuretics, nonsteroidal anti-inflammatory drugs, or anticoagulants. The percentage of patients with other antacids (sucralfate or amphojel suspension) was higher in PPI ever users than in PPI never users (17.9% vs. 3.1%, P = 0.002). The interval between the initial and follow-up brain MRI was also longer in PPI ever users than in PPI never users (mean 51.2±17.5 months vs. 42.7±20.2 months, P = 0.030). Over the follow-up periods, PPI users had more frequent osteopenia and osteoporosis than PPI never users (25.6% vs. 7.1%, P = 0.007) (S1 Table).

**Table 1:** Baseline characteristics of the included patients.

	PPI ever user (N = 39, 28.5%)	PPI never user (N = 98, 71.5%)	P-value
**Age, mean (SD), year**	65.6 ± 6.1	67.1 ± 9.1	0.002
**Male, n (%)**	12 (30.8)	42 (42.9)	0.246
**Hypertension, n (%)**	24 (61.5)	45 (45.9)	0.130
**Diabetes mellitus, n (%)**	3 (7.7)	20 (20.4)	0.081
**Dyslipidemia, n (%)**	15 (38.5)	33 (33.7)	0.692
**Previous stroke/TIA, n (%)**	6 (15.3)	26 (26.5)	0.164
**Coronary heart disease, n (%)**	7 (17.9)	10 (10.2)	0.253
**Laboratory parameters**			
** Creatinine, mean (SD), mg/dL**	0.99 ± 0.67	1.08 ± 1.11	0.485
**Co-prescription (except antacid)**			
** Calcium channel blocker, n (%)**	15 (38.5)	23 (23.5)	0.092
** ACEi/ARB, n (%)**	7 (17.9)	15 (15.3)	0.797
** Diuretics, n (%)**	3 (7.7)	9 (9.2)	1.000
** NSAID, n (%)**	12 (30.8)	20 (20.4)	0.263
** Any antiplatelet, n (%)**	19 (48.7)	29 (29.6)	0.034
** Anticoagulant, n (%)**	3 (7.7)	2 (2.0)	0.139
**Co-prescription (antacid)**			
** H2-receptor blocker, n (%)**	8 (20.5)	16 (16.3)	0.620
Others*, n (%)	7 (17.9)	3 (3.1)	0.002

Abbreviation: PPI, proton pump inhibitor; SD, standard deviation; TIA, transient ischemic attack; ACEi, angiotensin-converting enzyme inhibitors; ARB, angiotensin receptor blocker; NSAID, nonsteroidal anti-inflammatory drug; Others* include sucralfate, magnesium hydroxide, aluminium hydroxide, polaprezinc, and revaprazan.

### Factors associated with progression of WMH and CMB

**Fig 1:**
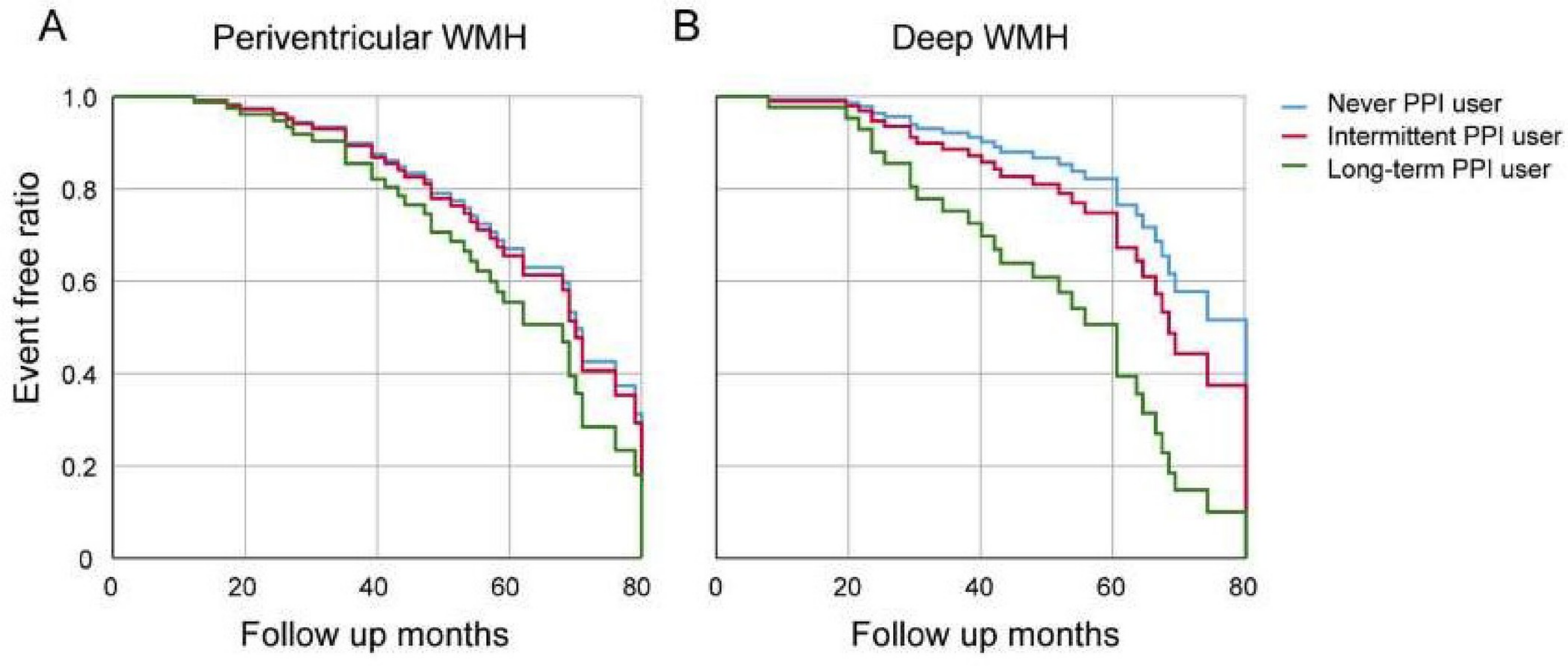
Multivariate Cox regression for progression of Fazekas score of white matter hyperintensity with the time since baseline as a dependent variable with proton pump inhibitor dose. The progression of periventricular WMH was not different among PPI never users, intermittent users, or long-term users (A). However, long-term PPI users had more progression of deep WMH compared to PPI intermittent users or PPI never users. Adjusted covariates were age, history of hypertension, diabetes, dyslipidemia, previous stroke or transient ischemic attack, coronary heart disease, and use of antiplatelet agents. Abbreviations: WMH, white matter hyperintensities; PPI, proton pump inhibitor.

The information on the baseline and follow-up Fazekas score (periventricular and deep) and CMB are presented in S2 Table. Baseline Fazekas scores (median 1 vs. median 1) and numbers of CMB (median 0 vs. median 0) did not differ between the two groups. Over the follow-up period, the Fazekas score (both periventricular and deep) was aggravated to a median of 2 in PPI ever users. However, the median numbers of CMB did not change between the two groups. To determine the dose-response relationship between PPI use and the progression of WMH or CMB, PPI users were further stratified into two groups: long-term users (n = 15, 38.5%) and PPI intermittent users (n = 24, 61.5%). Univariate Cox regression analysis showed that PPI use was not associated with Fazekas score progression in periventricular WMH (Table 2). However, PPI use was significantly associated with deep WMH in univariate Cox regression analysis (HR 2.891, 95% CI 1.210–6.909, P = 0.017), although it was not statistically significant in the multivariate analysis (Table 2). A dose-response relationship was assessed using univariate analysis and showed that intermediate and long-term PPI users had a higher risk of the progression of deep WMHs compared to PPI never users (HR 2.699, 95% CI 1.075–6.774, P = 0.034; HR 3.823, 95% CI 1.001–14.617, P = 0.048, respectively; P for trends, <0.001). However, multivariate Cox regression analysis showed that only long-term PPI use was associated with the progression of Fazekas score at deep WMH (HR 3.453, 95% 1.027–9.475, P = 0.045) after adjusting for confounders (Table 2, Fig 1). There was no association between PPI use and the progression of CMBs (Table 3).

**Table 2:** Factors associated with the progression of Fazekas score of white matter hyperintensities.

	Periventricular WMH				Deep WMH			
	Crude HR (95% CI*)	P-value	Adjusted HR (95% CI*)	P-value	Crude HR (95% CI*)	P-value	Adjusted HR (95% CI*)	P-value
**PPI use**	1.868 (0.834–4.184)	0.129	2.139 (0.893–5.126)	0.088	2.891 (1.210–6.906)	0.017	2.036 (0.744–5.572)	0.167
** Never user**	1 (References)		1 (References)		1 (References)		1 (References)	
** Intermediate user**	1.947 (0.838–4.524)	0.121	2.198(0.890–5.427)	0.088	2.699 (1.075–6.774)	0.034	1.719 (0.773–3.822)	0.184
** Long term user**	1.818 (0.402–8.222)	0.437	1.787(0.272–11.715)	0.545	3.823 (1.001–14.617)	0.048	3.453 (1.027–9.475)	0.045
**Age at initial imaging**	0.995 (0.944–1.048)	0.837	0.990(0.934–1.049)	0.733	0.988 (0.935–1.043)	0.656	0.952 (0.872–1.038)	0.265
**Hypertension**	1.038 (0.471–2.288)	0.926	1.165(0.483–2.811)	0.735	1.818 (0.776–4.260)	0.169	1.666 (0.397–6.983)	0.581
**Diabetes mellitus**	0.827(0.306–2.239)	0.709	0.637(0.208–1.957)	0.431	0.514(0.150–1.761)	0.290	2.836 (0.018–45.283)	0.371
**Dyslipidemia**	1.501 (0.661–3.410)	0.332	1.723(0.678–4.379)	0.253	1.508 (0.630–3.608)	0.357	0.804 (0.161–4.005)	0.804
**Previous stroke/TIA**	1.643 (0.651–4.144)	0.293	2.649(0.889–7.888)	0.080	0.797 (0.235–2.709)	0.717	1.777 (0.356–8.867)	0.910
**Coronary heart disease**	0.983 (0.294–3.290)	0.978	0.697(0.184–2.640)	0.595	2.206 (0.812–5.996)	0.121	1.128 (0.138–9.190)	0.910
**Use of antiplatelet agents**	0.645 (0.266–1.562)	0.331	0.983(0.408–2.366)	0.969	0.624 (0.245–1.591)	0.323	1.430 (0.285–7.179)	0.664

WMH, white matter hyperinitensities; HR, hazard ratio; CI, confidence interval; PPI, proton pump inhibitor; TIA, transient ischemia attack

**Table 3:** Factors associated with the progression of microbleeds.

	Unadjusted HR (95% CI*)	P-value
**PPI use**	1.368 (0.321–5.831)	0.672
** Never use**	1 (References)	
** Intermediate use**	1.603 (0.176–14.637)	0.676
** Long term use**	3.473 (0.381–31.673)	0.270
**Age at initial imaging**	0.952 (0.872–1.038)	0.265
**Hypertension**	1.666 (0.397–6.983)	0.581
**Diabetes**	2.836 (0.018–45.283)	0.371
**Dyslipidemia**	0.804 (0.161–4.005)	0.804
**Previous stroke/TIA**	1.777 (0.356–8.867)	0.910
**Coronary heart disease**	1.128 (0.138–9.190)	0.910
**Use of antiplatelet agents**	1.430 (0.285–7.179)	0.664

*Abbreviation: HR, hazard ratio; CI, confidence interval; PPI, proton pump inhibitor; MRI, magnetic resonance imaging; TIA, transient ischemia attack

## Discussion

The present study showed that PPI use was associated with the progression of deep cerebral WMHs. In addition, the effect was more robust among long-term PPI users (more than six months) compared to PPI intermittent users or PPI never users, suggesting that cumulative effects of PPI may exist.

Long-term use of PPI was associated with an increase in myocardial infarction [6, 18]. Among the suggested mechanisms, drug interaction between clopidogrel and PPIs, especially omeprazole, may play a role [19–21]. Given that clopidogrel is a prodrug and is converted to its active form by cytochrome P450, the coadministration of clopidogrel with omeprazole decreases the maximum concentration of clopidogrel by 40%. However, in our study, only 16% of patients used clopidogrel, and cerebral SVD was aggravated in PPI users regardless of clopidogrel use. Therefore, we suggest that the progression of SVD is not merely due to the interaction between clopidogrel and PPIs. Previous reports also showed that PPI use was also associated with a higher risk of incident chronic kidney disease (CKD) [22]. Given that CKD shares the same risk factors with cerebral SVD and is considered a systemic surrogate marker for cerebral SVD, long-term PPI use may accelerate systemic and cerebral vascular endothelial senescence [23]. Administration of PPI is known to increase the asymmetric dimethylarginine (ADMA), which leads to enhanced vascular resistance and promotes inflammation, or may reduce telomere length or impede the effect of nitric oxide on vascular endothelium [7, 22].

The most crucial finding in this study was that the progression of deep WMH, not periventricular WMH and CMB, was associated with PPI use. The degree of SVD increases with aging, as shown in S2 Table. To distinguish the effect of aging on the progression of SVD, we performed a Cox regression analysis. As shown in Fig 1, PPI use had a dose-dependent effect on the progression of periventricular and deep WMH. However, the statistically distinctive feature was the progression of deep WMHs among the PPI long-term users, suggesting cumulative dose dependency. Progression of periventricular WMH is highly associated with alterations in age-related cerebrospinal fluid dynamics. In contrast, deep WMH is more affected by endothelial activation or fibrohyalinosis of the arterioles, considered ischemic insults [24]. Similarly, a recent study showed that deep WMH, but not periventricular WMH, was associated with hypertension and vascular dementia [25]. Periventricular WMH may be more vulnerable to hypoperfusion, suggesting that the causes of the two WMHs may be different [10, 24–26].

Although CMBs are regarded as a surrogate marker for SVD, the factors associated with an aggravation of CMBs are amyloid microangiopathy, hypertension, or low cholesterol levels, which is different from ischemia itself [11]. Therefore, we believe that this may explain why we could not observe any association between PPI use and the progression of CMBs. In the results, we did not find a direct association between the progression of CMB and hypertension, which could be due to the small number of patients.

This study has several limitations. First, this is a retrospective observational study on patients who had repeated brain imaging. Therefore, there is a chance of selection bias, and the results need to be interpreted with caution. Second, the number of patients was relatively small. Third, we used the government-monitored drug utilization review (DUR) system for screening and cross-checking the prescribed medications. This system might not be perfect in identifying patients who were prescribed PPIs from other hospitals, even though we do not think that the chance is high. Fourth, although we categorized PPI ever users as PPI intermittent users and PPI long-term users based on the prescription information from the DUR system, we do not have any information on drug adherence. Fifth, we did not compare the effect of PPIs on WMH with that of anti-acids, which requires further study. Sixth, we did not exclude patients with a previous history of ischemic stroke or TIA but did those who had within 90 days. Therefore, a potentially negative effect of PPIs on WMHs might be overestimated because the patients with a previous history of ischemic stroke or TIA are potentially more vulnerable to further damages. In real practice, one of the reasons for prescribing PPIs is to prevent antiplatelet related gastrointestinal bleeding. In this study, 56.4% of patients on PPIs are also taking antiplatelet agents or anticoagulants. Therefore, we thought that analyzing the effect of PPIs on WMHs in stroke-naïve patients also has a limitation in applying the results in real practice. The effect of PPIs on WMH might be different in patients with or without prior history of stroke or TIA. This requires further study. Finally, we did not analyze a differential effect of PPIs on endothelial cells. Further studies are needed to understand the intrinsic activity of individual PPIs.

In conclusion, the long-term use of PPI for more than six months was associated with the progression of deep WMH. Given that progression of cerebral SVD is associated with ischemic stroke or cognitive decline, clinicians should consider the possible negative effects of long-term PPI use.

## Supporting information

S1 Fig Selection of the patients through the inclusion and exclusion criteria. * Abbreviation: MRI, magnetic resonance imaging; MRA, magnetic resonance angiography; PPI, proton pump inhibitor.
(TIF)

S1 Table Other adverse outcomes among the subjects. Abbreviation: PPI, proton pump inhibitor.
(DOCX)

S2 Table Change of white matter hyperintensities and cerebral microbleeds among the subjects. PPI, proton pump inhibitor; IQR, interquartile range.
(DOCX)

S1 Data (XLSX)
