# Supplementary figures and images for: Use of proton pump inhibitor may be associated with progression of cerebral small vessel disease
Source: PLoS One. 2022 Dec 21;17(12):e0279257. doi: 10.1371/journal.pone.0279257 (PMC9770424; doi:10.1371/journal.pone.0279257)

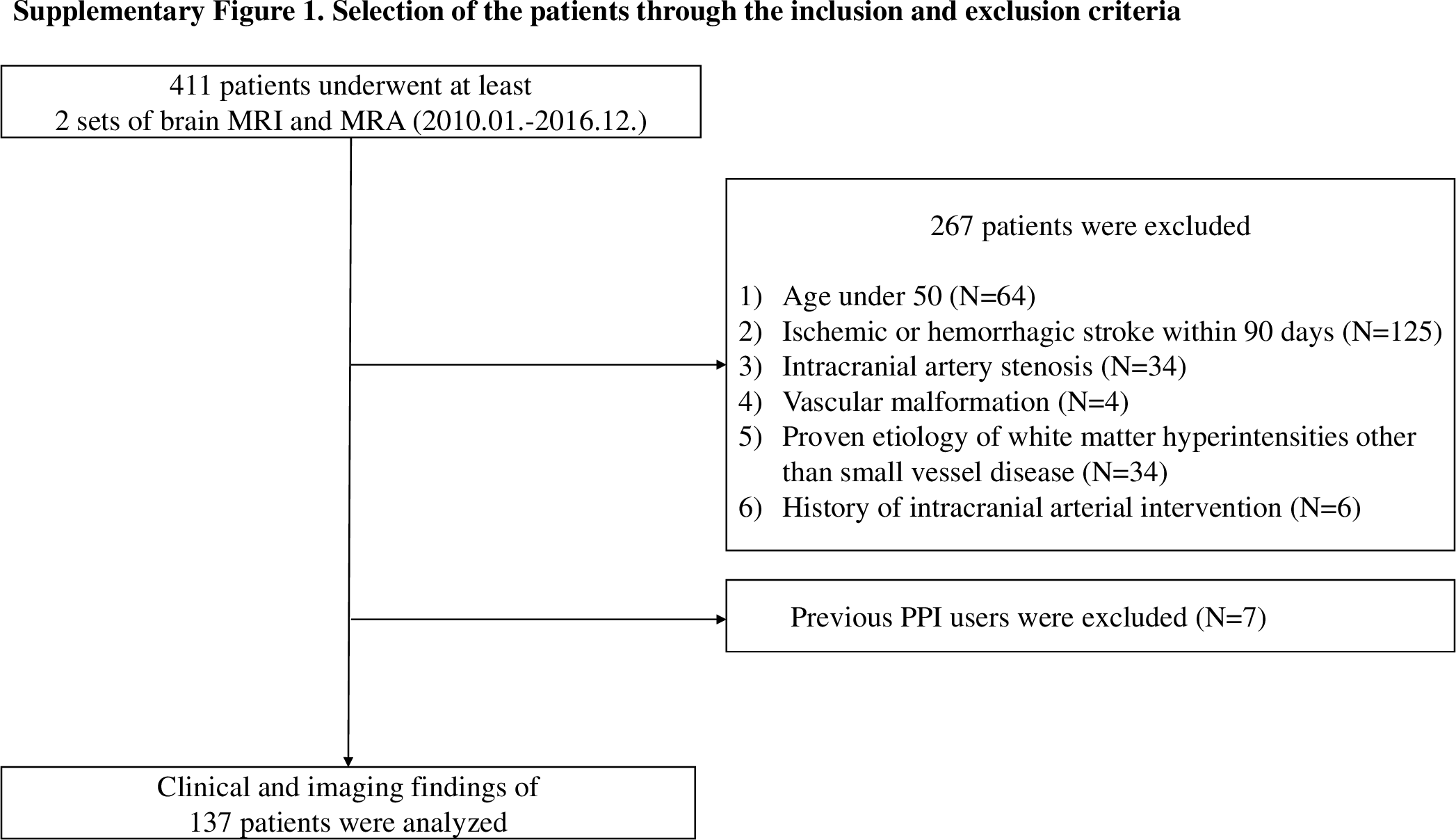

Supplement: S1 Fig — * Abbreviation: MRI, magnetic resonance imaging; MRA, magnetic resonance angiography; PPI, proton pump inhibitor. (TIF) [file pone.0279257.s001.tif]
