# Supplementary material for: Use of proton pump inhibitor may be associated with progression of cerebral small vessel disease
Source: PLoS One. 2022 Dec 21;17(12):e0279257. doi: 10.1371/journal.pone.0279257 (PMC9770424; doi:10.1371/journal.pone.0279257)
Supplement: S1 Table — Abbreviation: PPI, proton pump inhibitor. (DOCX) [file pone.0279257.s002.docx]

**Supplementary table 1. Other adverse outcomes among the subjects**

|  | **PPI ever user**  **(N=39, 28.5%)** | **PPI never user**  **(N=98, 71.5%)** | **P-value** |
| --- | --- | --- | --- |
| **Pneumonia, n (%)** | 3 (7.7) | 8 (8.2) | 1.000 |
| **Fracture, n (%)** | 3 (7.7) | 1 (1.0) | 0.070 |
| **Osteopenia/Osteoporosis, n (%)** | 10 (25.6) | 7 (7.1) | 0.007 |

Abbreviation : PPI, proton pump inhibitor
