# Supplementary material for: Use of proton pump inhibitor may be associated with progression of cerebral small vessel disease
Source: PLoS One. 2022 Dec 21;17(12):e0279257. doi: 10.1371/journal.pone.0279257 (PMC9770424; doi:10.1371/journal.pone.0279257)
Supplement: S2 Table — PPI, proton pump inhibitor; IQR, interquartile range. (DOCX) [file pone.0279257.s003.docx]

**Supplementary table 2. Change of white matter hyperintensities and cerebral microbleeds among the subjects**

|  | **PPI ever user**  **(N=39, 28.5%)** | **PPI never user**  **(N=98, 71.5%)** | **P-value** |
| --- | --- | --- | --- |
| **Baseline periventricular Fazekas score, median (IQR)** | 1 (1-2) | 1 (1-2) | 0.293 |
| **Baseline deep Fazekas score, median (IQR)** | 1 (0-2) | 1 (1-2) | 0.214 |
| **Baseline cerebral microbleeds count, median (IQR)** | 0 (0-0) | 0 (0-0) | 0.442 |
| **Follow-up periventricular Fazekas score, median (IQR)** | 2 (1-2) | 2 (1-2) | 0.971 |
| **Follow-up deep Fazekas score, median (IQR)** | 2 (1-2) | 1 (1-2) | 0.516 |
| **Follow-up cerebral microbleeds count, median (IQR)** | 0 (0-0) | 0 (0-0) | 0.838 |

PPI, proton pump inhibitor; IQR, interquartile range
